# Fabrication of attapulgite/magnetic aminated chitosan composite as efficient and reusable adsorbent for Cr (VI) ions

**DOI:** 10.1038/s41598-021-96145-6

**Published:** 2021-08-16

**Authors:** Abdelazeem S. Eltaweil, Eman M. Abd El-Monaem, Mohamed S. Mohy-Eldin, Ahmed M. Omer

**Affiliations:** 1 Chemistry Department, Faculty of Science, Alexandria University, Alexandria, Egypt; 2 Polymer Materials Research Department, Advanced Technology and New Materials Research Institute (ATNMRI), City of Scientific Research and Technological Applications (SRTA-City), New Borg El-Arab City, P. O. Box: 21934, Alexandria, Egypt

**Keywords:** Chemistry, Materials science

## Abstract

An efficient composite was constructed based on aminated chitosan (NH_2_Cs), attapulgite (ATP) clay and magnetic Fe_3_O_4_ for adsorptive removal of Cr(VI) ions. The as-fabricated ATP@Fe_3_O_4_-NH_2_Cs composite was characterized by Fourier Transform Infrared Spectroscopy (FTIR), Thermal Gravimetric Analyzer (TGA), Scanning Electron Microscope (SEM), Zeta potential (ZP), Vibrating Sample Magnetometer (VSM), Brunauer–Emmett–Teller method (BET) and X-ray photoelectron spectroscope (XPS). A significant improve in the adsorption profile was established at pH 2 in the order of ATP@Fe_3_O_4_-NH_2_Cs(1:3) > ATP@Fe_3_O_4_-NH_2_Cs(1:1) > ATP@Fe_3_O_4_-NH_2_Cs(3:1) > Fe_3_O_4_-NH_2_Cs > ATP. The maximum removal (%) of Cr(VI) exceeded 94% within a short equilibrium time of 60 min. The adsorption process obeyed the pseudo 2nd order and followed the Langmuir isotherm model with a maximum monolayer adsorption capacity of 294.12 mg/g. In addition, thermodynamics studies elucidated that the adsorption process was spontaneous, randomness and endothermic process. Interestingly, the developed adsorbent retained respectable adsorption properties with acceptable removal efficiency exceeded 58% after ten sequential cycles of reuse. Besides, the results hypothesize that the adsorption process occurs via electrostatic interactions, reduction of Cr(VI) to Cr(III) and ion-exchanging. These findings substantiate that the ATP@Fe_3_O_4_-NH_2_Cs composite could be effectively applied as a reusable adsorbent for removing of Cr(VI) ions from aqueous solutions.

## Introduction

Indeed, the recent prosperity of industries has obvious positive impacts on economic growth; however, disposal of the industrial effluents into the water bodies without reasonable treatment can undoubtedly eradicate mankind^1,2^. One of the most toxic pollutants that pose jeopardy to human health as well as the entire environment is hexavalent chromium (Cr(VI)). Therefore, Cr(VI) has been deemed to be carcinogenic, mutagenic and displayed higher toxicity due to its enormously mobile in the surroundings^3,4^. Subsequently, the World Health Organization has authorized that the maximum limit of Cr(VI) in potable water should not exceed 0.05 mgL^−1^^5,6^. Despite these mentioned risks, there are number of significant industries mainly based on Cr(VI) including steel, textile, dyeing, cement, electroplating and leather tanneries^7,8^. Therefore, several researches have been focused on the removal of Cr(VI) ions from their aqueous solutions via diverse techniques including coagulation^9^, chemical precipitation^10^, adsorption^11,12^, membrane separation^13^, catalysis^14^, ion exchange^15^ and electrodialysis^16^. Principally, there are strict criteria to select the appropriate removal technique such as low energy consumption, process simplicity, renewability and the low operational cost^17^. Accordingly, adsorption can be considered as the most preferred technique to remove Cr (VI) from aqueous solution^18,19^. Consequently, a plethora of the adsorbents such as activated carbon, clay materials, polymers, natural products, and metal/mixed oxide nanoparticles have been used for the removal of Cr(VI) from wastewater^20,21^.

Chitosan (Cs) is a cationic polysaccharide polymer that is easily obtained via N-deacetylation of chitin, the essential component of the exoskeleton of crustaceans like shrimp, fungi, crab and insects^22,23^. Owing to its unparalleled merits such as biocompatibility, polyelectrolyte properties, recyclability, hydrophilicity, biodegradability and adhesion properties, chitosan has a significant deal of interest as an efficient cationic adsorbent for removal of heavy metals, pharmaceutical pollutants and organic dyes from their aqueous solutions^24,25^. Besides, the presence of the chemically active groups in the chitosan structure such as amino and hydroxyl groups enable the formation of chitosan-derivatives with worthy properties by Schiff base formation, grafting, carboxymethylation, and amination^26–28^. For instance, aminated chitosan is a newly-established chitosan derivative with extra amine groups, which is expected to enrich the adsorption characteristics of the native chitosan^29,30^. Although all these features of chitosan, it possesses serious drawbacks including low adsorption kinetic, low surface area, high tendency to agglomerate, poor mechanical strength and low adsorption capacity^31,32^.

Incorporation of clays into chitosan matrices is a feasible solution to overcome its flaws since clays have great features; good adsorptive properties, low cost, high thermal stability, high surface area and special catalytic activity^33–35^. Amongst these clays, attapulgite is a subset of hydrous magnesium aluminum silicate with concrete features including hydrophilicity, non-toxicity, low price, abundant resources as well as its high surface area and high porosity^36,37^. Therefore, attapulgite has been vastly utilized in vital fields such as agriculture, catalysis, anticorrosion and wastewater treatment^38–40^.

Based on aforementioned interests, an attempt was made in this study to fabricate a new adsorbent composite for efficient adsorptive removal of Cr(VI) ions from their aqueous solutions with a highly adsorption performance and better recyclability. Taking advantages of NH_2_Cs derivative and ATP clay as well as to allow their beneficial adsorption features to be combined. Herein, ATP@Fe_3_O_4_-NH_2_Cs magnetic composite was successfully synthetized and well-characterized using several analyses tools. Moreover, the aptitude adsorption of the developed adsorbent toward of Cr(VI) ions was achieved using a batch adsorption technique under several studied conditions. Furthermore, isotherms, kinetics and thermodynamics studies were thoroughly studied. Besides, the ability of the developed adsorbent composite to be reuse for ten consecutive adsorption cycles was also examined.

## Experimental section

### Materials

Chitin (degree of acetylation = 0.94) was purchased from Daejung (Korea), Potassium dichromate (Assay ≥ 99%), *Para*-benzoquinone (PBQ; 99%), Iron chloride hexahydrate (≥ 99%) and Ferrous chloride tetrahydrate (≥ 99%) were delivered from Sigma-Aldrich (Germany). Ethylenediamine (EDA; 99%), Glutaraldehyde (25%) and Sodium hydroxide (98%) were brought from Aladdin Industrial Corporation (China). Attapulgite (ATP) was supplied from the Huaiyuan Mining Industry Co., Ltd. (China). Hydrochloric acid (37%), Ammonium hydroxide (99%) and Acetic acid (98%) were acquired from Loba Chemie (India).

### Synthesis of magnetite Fe_3_O_4_

Magnetic Fe_3_O_4_ was made-up by co-precipitation technique^41^. Exactly, FeCl_3_·6H_2_O (0.092 mol) and FeCl_2_·4H_2_O (0.046 mol) were dissolved into 300 mL distilled H_2_O water under N_2_ atmosphere. After that, ammonia solution (25%) was slowly added to the reaction solution until pH reaches 9 and then the solution kept under magnetic stirring for 80 min at 70 °C. Ultimately, the formed particles were separated utilizing an external magnet, washed with ethanol and ultimately dried at 60 °C for 12 h.

### Synthesis of aminated chitosan (NH_2_Cs ) derivative

NH_2_Cs was synthesized according to the authors preceding work with a slight modification^42^.Transformation of chitin to NH_2_Cs was achieved via three main steps. The first step involves the activation of –OH^−^ groups of chitin in which 8 g of chitin was soaked into PBQ solution (6.9 mM; pH 10) which acts as an activator agent. The reaction mixture was conducted under continuous stirring for 6 h at 60 °C. The resultant activated chitin was washed with distilled H_2_O to remove the excess of PBQ molecules. The second step includes the formation of amino-chitin, since, and followed by dispersion in EDA solution (6.9 mM) for 6 h under constant stirring at 60 °C. The obtained aminated chitin was separated and washed several times using distilled H_2_O to remove the unreacted EDA molecules. Finally; the third step involves deacetylation of aminated chitin which was achieved by immersing it in NaOH (50%) solution for 22 h under magnetic stirring at 140 °C. The gotten aminated chitosan (NH_2_Cs) was filtrated, washed with distilled H_2_O and dried at 60 °C.

### Fabrication of ATP@Fe_3_O_4_-NH_2_Cs composite

A specific amount of NH_2_Cs was dissolved into 20 mL of acetic acid (2%; v/v) aqueous solution under ultrasonic stirring for 45 min. Next, 0.02 g of Fe_3_O_4_ was slowly added into NH_2_Cs solution, and then kept under vigorous stirring for 90 min until the reaction solution became totally homogenous. Then after, an appropriate amount of ATP clay was added to the reaction mixture, and followed by adding 4 mL of glutaraldehyde (25%; v/v) solution. The composite mixture was left under continuous stirring at 60 °C for another 90 min. lastly, the resultant composite was separated, washed with ethanol and left overnight for drying at 45 °C. The ATP@Fe_3_O_4_-NH_2_Cs composite was prepared with different weight ratios of ATP and Fe_3_O_4_-NH_2_Cs composite namely; ATP@Fe_3_O_4_-NH_2_Cs (1:3), ATP@Fe_3_O_4_-NH_2_Cs (1:1) and ATP@Fe_3_O_4_-NH_2_Cs (3:1), respectively.

**Figure 1:**
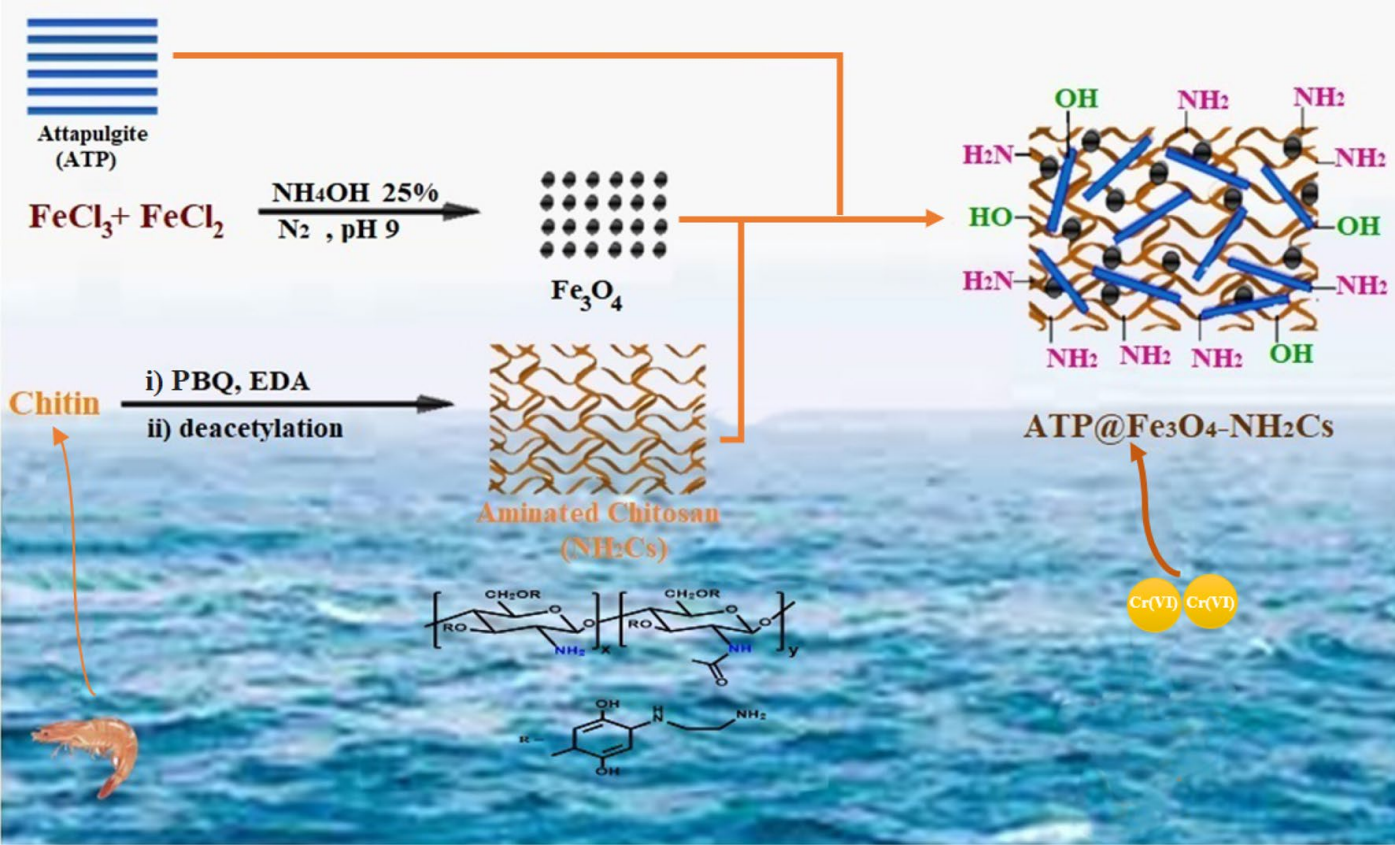
A schematic representation for the fabrication process of ATP@Fe_3_O_4_-NH_2_Cs magnetic composite.

A schematic representation for the fabrication of ATP@Fe_3_O_4_-NH_2_Cs magnetic composite was depicted in Fig. 1.

### Characterization

To investigate the surface morphologies of developed ATP@Fe_3_O_4_-NH_2_Cs composite as well as NH_2_Cs and ATP clay a Scanning Electron Microscope (SEM; Joel Jsm 6360LA, Japan) was employed under a voltage potential of 20 kV. The examined samples were placed on aluminum stumps and coated with a thin layer of gold via a sputter coating system. The thermal stability was examined under nitrogen atmosphere by Thermal Gravimetric Analyzer (TGA; Shimadzu-50, Japan), while the temperature was raised from 10 to 700 °C at constant heating rate of 20 °C/min and flow rate of 40 mL/min. In addition, the chemical composition of ATP@Fe_3_O_4_-NH_2_Cs composite was explored by Fourier Transform Infrared Spectroscopy (FTIR; Shimadzu-8400 S, Japan), while the absorbance was scanned in the wavenumber range 500–4000 cm^–1^. Besides, a vibrating Sample Magnetometer (VSM-8600, Lake Shore Cryotronics, Inc., USA) was utilized for evaluating the magnetic property, while Zeta potential (Malvern, UK) was utilized to determine the surface charge. X-ray photoelectron spectroscope (XPS, Axis Ultra DLD, Shimadzu, Japan) was employed for examining the elemental-surface composition of the developed adsorbent. Furthermore, the specific surface area was measure using Brunauer–Emmett–Teller method (BET; Beckman Coulter, SA3100, USA).

### Adsorption studies

Batch experiments were executed for evaluating the adsorption profile of ATP@Fe_3_O_4_-NH_2_Cs composite. An accurate 0.01 g of ATP@Fe_3_O_4_-NH_2_Cs composite was soaked into 20 mL of Cr (VI) solution with different concentration ranging from 50 to 200 mg/L at a constant stirring speed (200 rpm min^−1^). To optimize pH medium, pH of Cr (VI) solution was adjusted ranging from 1 to 8 by utilizing an aqueous solution of a strong acid and/or base. Moreover, the effect of ATP@Fe_3_O_4_-NH_2_Cs composite dosage onto adsorption of Cr (VI) was studied in the range 0.001–0.025 g, as well as the temperature effect, was studied in range 25–55 °C. After each experiment, the magnetic adsorbent was separated by an external magnet and the remaining concentration of Cr (VI) was detected via a spectrophotometer at λ_max_ = 540 nm. The adsorption capacity (q) and the removal percent (R%) were calculated from Eqs. 1 and 2, respectively.

1 $${\mathrm{q}}_{(\mathrm{mg}/\mathrm{g})}=\frac{{(\mathrm{C}}_{0}-{\mathrm{C}}_{\mathrm{t}}) \times \mathrm{V}}{\mathrm{W}}$$

2 $$\mathrm{R \%}=\frac{{\mathrm{C}}_{0}-{\mathrm{C}}_{\mathrm{t}}}{{\mathrm{C}}_{\mathrm{o}}} \times 100$$

where, C_o_ and C_t_, are the Cr(VI) initial concentration and its concentration at time t, respectively. While, V and W are the volume of Cr(VI) and the weight of ATP@Fe_3_O_4_-NH_2_Cs composite, respectively.

### Reusability test

From the economical point of view, the selection of an efficient adsorbent strongly depends on its recycling characteristic quality. Therefore, recyclability test was executed to assess the reuse aptitude for the ATP@Fe_3_O_4_-NH_2_Cs magnetic composite. In brief, the magnetic ATP@Fe_3_O_4_-NH_2_Cs composite was collected after completion the adsorption process by an exterior magnet, and followed by immersing in 25 mL of the desorption medium comprising of Methanol/NaCl solution mixture under stirring for 1 h. After complete the desorption process, ATP@Fe_3_O_4_-NH_2_Cs composite was separated magnetically for reuse for ten consecutive cycles.

All experiments were conducted in triplicate, and the results obtained were represented as the means corrected by standard deviation (± S.D.).

## Results and discussion

### Adsorbent characterization

#### FTIR

**Figure 2:**
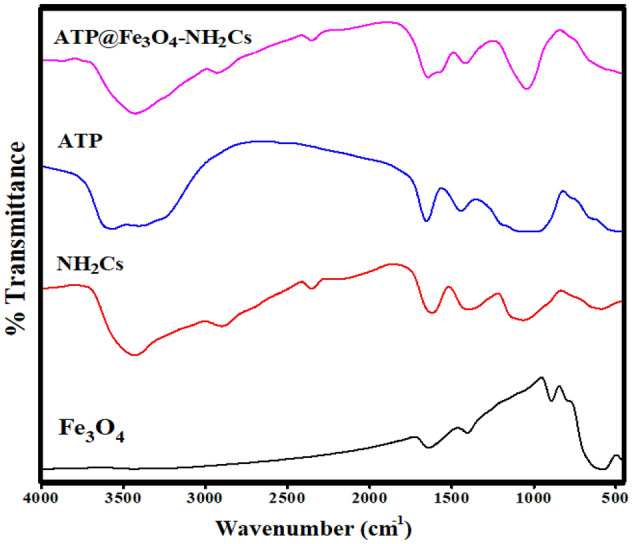
FTIR of Fe_3_O_4_, NH_2_Cs, ATP and ATP@Fe_3_O_4_-NH_2_Cs composite.

Figure 2 shows FTIR spectra of Fe_3_O_4_, NH_2_Cs, ATP and ATP@Fe_3_O_4_-NH_2_Cs composite. The spectrum of Fe_3_O_4_ reveals absorption broad at 3437 cm^−1^ which is ascribed to stretching vibration of –OH^−^ group^43^. In addition, the detected bands at 1639 and 892 cm^−1^ are assigned to –OH^−^ bending and vibrating modes, correspondingly^44^. Furthermore, the observed bands at 557 and 1405 cm^-1^ are related to Fe–O stretching^45^. FTIR spectrum of NH_2_Cs points out absorption bands at 2901, 2216 and 1619 cm^−1^ which correspond to CH_2_, COH stretching and N–H bending vibrations, respectively^46^. Besides, the broad bands at 3441 and 1062 cm^−1^ which are ascribed to stretching vibration of –OH^−^ and C–N groups, respectively. Additionally, there are two bands at 2907 and 1402 cm^−1^ which attributed to C–H stretching vibration and in-plane bending vibration, respectively^31^. The spectrum of ATP shows a peak at 3563 cm^-1^ belongs to M–OH bonds stretching vibration, where M; Si, Mg and Al. Also, bands at 3404 and 1653 cm^−1^ are related to vibrations of OH bending and OH stretching, respectively^36^. Moreover, the band at 484 cm^−1^ is ascribed to Si–O–Si bond binding vibration^39^. FTIR spectrum of ATP@Fe_3_O_4_-NH_2_Cs composite elucidates the fundamental peaks of the pristine materials (i.e. Fe_3_O_4_, NH_2_Cs and ATP), inferring the successful fabrication of ATP@Fe_3_O_4_-NH_2_Cs composite.

#### XPS

**Figure 3:**
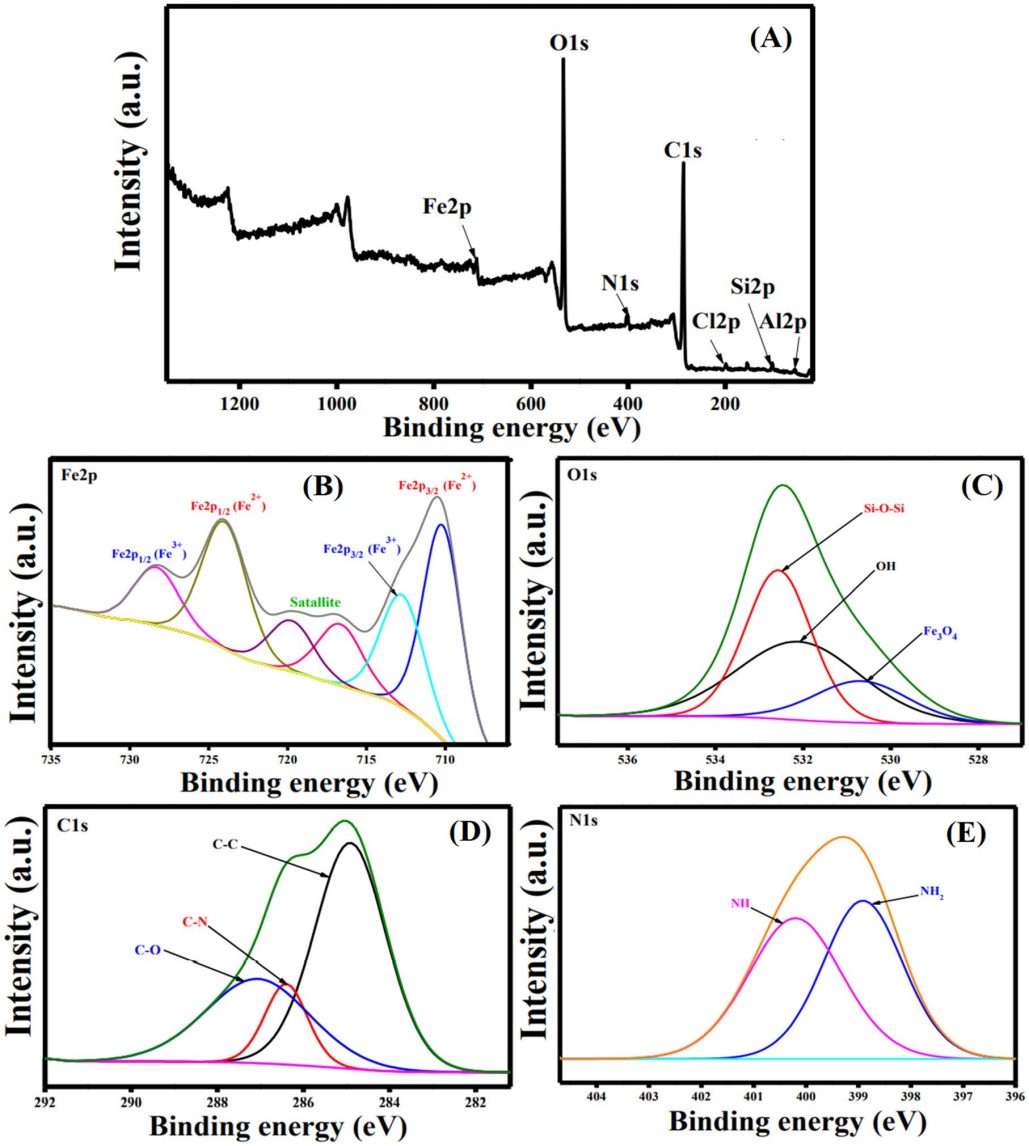
XPS spectra (**A**) wide scan of ATP@Fe_3_O_4_-NH_2_Cs composite, (**B**) Fe2p, (**C**) O1s, (**D**) C1s and (**E**) N1s.

The elemental composition of the as-fabricated ATP@Fe_3_O_4_-NH_2_Cs composite was scrutinized by XPS analysis. The wide-spectrum (Fig. 3A) illustrates the main elements of the composite; Al2p, Si2p, Cl2p, C1s, N1s, O1s and Fe2p at binding energy (BE) of 58.08, 103.15, 198.79, 286.35, 401.47, 533.04 and 712.18 eV, respectively^47^.The high-resolution spectrum of Fe2p (Fig. 3B) verifies that the as-fabricated composite contains Fe^2+^ (Fe 2p_3/2_: 710.17 eV, Fe 2p_1/2_: 724 eV and satellites: 716.66 and 719.77 eV) and Fe^3+^ (Fe 2p_3/2_: 712.72 eV,Fe 2p_1/2_: 728.3 eV)^48^. Moreover, the high-resolution spectrum of O1s (Fig. 3C) shows a peak at BE of 530.63 eV which is ascribed to the oxygen atoms in Fe_3_O_4_ lattice. Furthermore, the peak at BE of 532.18 eV is related to OH groups, while the peak at BE of 532.59 is due to Si–O–Si of ATP clay^49^. Besides, the high-resolution of N1s (Fig. 3D) demonstrates the distinguishing peaks of NH and NH_2_ groups at BE of 400.26 and 398.97 eV, respectively. The high-resolution of C1s (Fig. 3E) reveals three peaks at BE of 287.06 (for C–O), 286.4 (for C–N) and 284.91 (for C–C), respectively^50^.

#### SEM

**Figure 4:**
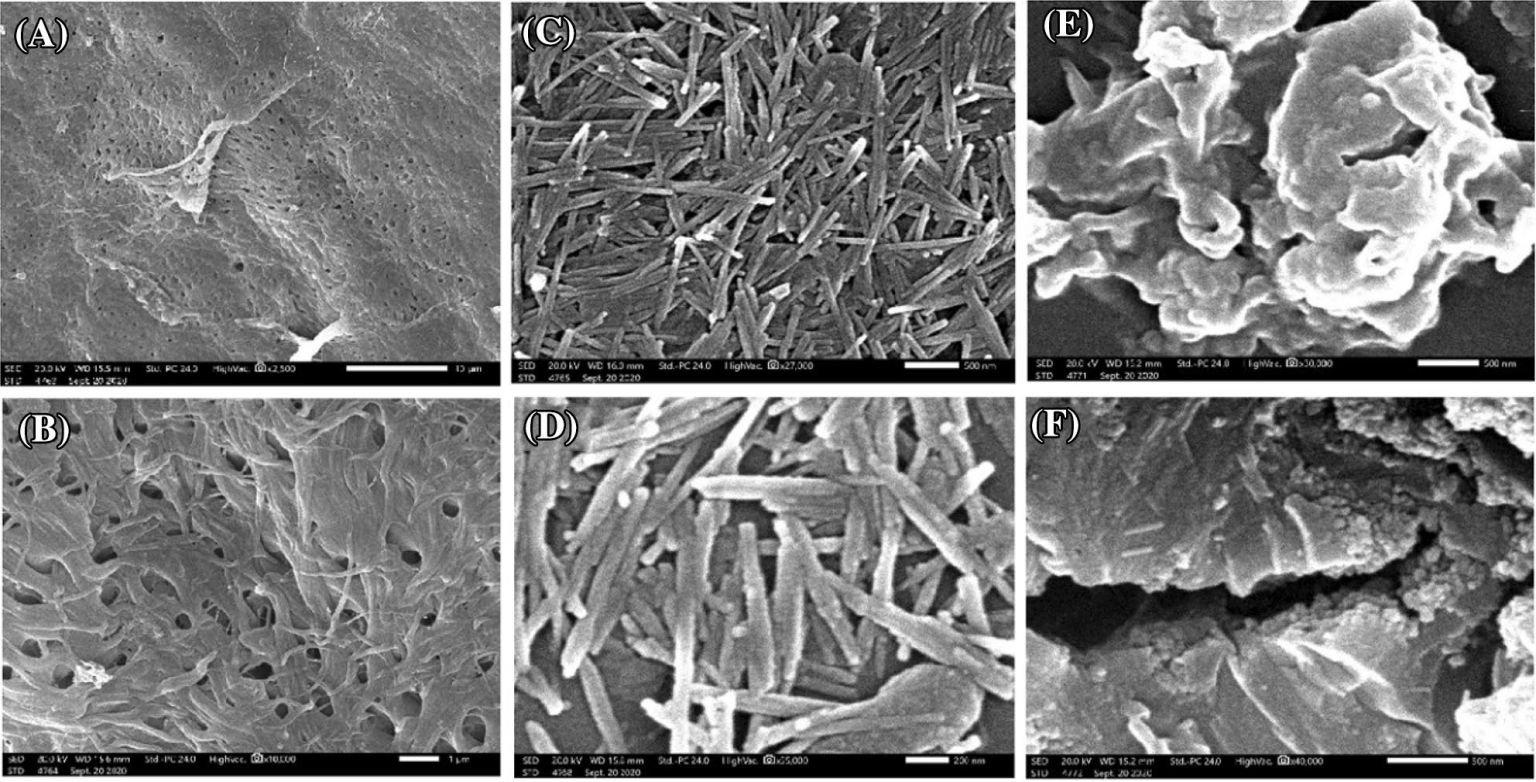
SEM of (**A**,**B**) NH_2_Cs, (**C**,**D**) ATP and (**E**,**F**) ATP@Fe_3_O_4_-NH_2_Cs composite**.**

SEM images (Fig. 4A,B) depicted that NH_2_Cs surface looks like a spongy network with open and interconnected pores. While, SEM images (Fig. 4C,D) showed that ATP clay has a rod-like structure in nano size. On the other hand, SEM images (Fig. 4E,F) revealed the layered structure of ATP@Fe_3_O_4_-NH_2_Cs composite with some interlayer spaces. Furthermore, brightness spots in some regions was noticed confirming the existence of exfoliation of ATP particles in the NH_2_Cs matrix^51^.

#### TGA

**Figure 5:**
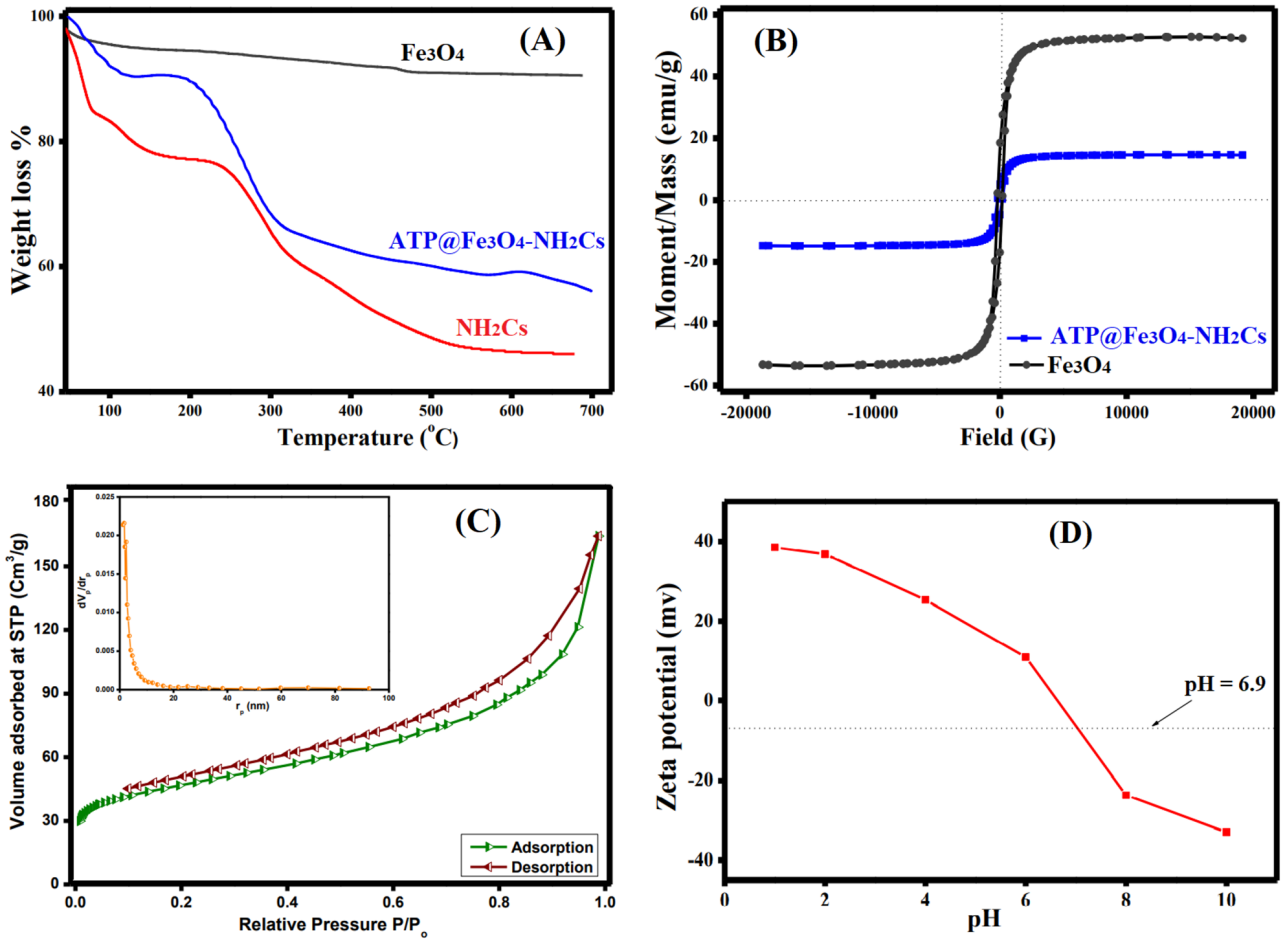
(**A**) TGA of Fe_3_O_4,_ NH_2_Cs and ATP@Fe_3_O_4_-NH_2_Cs composite, (**B**) VSM of Fe_3_O_4_ and ATP@Fe_3_O_4_-NH_2_Cs composite (**C**) N_2_ adsorption/desorption isotherm and pore size distribution and (**D**) ZB ATP@Fe_3_O_4_-NH_2_Cs composite.

Thermal stability of the fabricated samples was scrutinized using TGA analysis at the temperature range from 45 to 700 °C (Fig. 5A). TGA curve of pure Fe_3_O_4_ illustrates a slight weight loss of about 5.75% from 45 to 450 °C which is ascribed to the removal of moisture^52^. Moreover, TGA curve of NH_2_Cs depicts three weight loss stages; the first stage was recorded up to 150 °C which may be attributed to the evaporation of the adsorbed water, while the second one was recorded up to 350 °C which may be due to the dehydration of the saccharide rings and depolymerization of NH_2_Cs^53^. Besides, the third weight loss that was recorded up to 600 °C elucidates the complete decomposition of NH_2_Cs^54^. It is apparent from TGA curve of ATP@Fe_3_O_4_-NH_2_Cs composite that the combination of NH_2_Cs with ATP and Fe_3_O_4_ ameliorates its thermal behavior at which the total weight loss of NH_2_Cs and ATP@Fe_3_O_4_-NH_2_Cs composite was 53.95 and 43.92%, respectively.

#### VSM

Figure 5B represents the magnetic behaviors of the fabricated Fe_3_O_4_ and ATP@Fe_3_O_4_-NH_2_Cs composite. The magnetization loops of both Fe_3_O_4_ and ATP@Fe_3_O_4_-NH_2_Cs composite reveal a ferromagnetic behavior as the coericivity values were 198.76 and 90.68 G, respectively. Moreover, the saturation magnetization of Fe_3_O_4_and ATP@Fe_3_O_4_-NH_2_Cs composite were 52.31 and 14.53 emu/g, respectively. This expected decrease in the saturation magnetization of Fe_3_O_4_ may be due to the shielding of polymer–clay layer^55,56^. However, the declined saturation magnetization of ATP@Fe_3_O_4_–NH_2_Cs composite, itis sufficient enough to provide a perfect magnetic separation.

#### BET

Figure 5C depicts the N_2_ adsorption/desorption hysteresis loop and the pore size distribution of ATP@Fe_3_O_4_-NH_2_Cs composite. The hysteresis loop reveals microporous structure of ATP@Fe_3_O_4_-NH_2_Cs composite at which the relatively low pressure (P/P_o_ < 0.05 atm) significantly increased. Besides, the BET isotherm represents type IV with H4 hysteresis loop, indicating the existence of mesoporous. Moreover, the specific surface area and the total pore diameter were 164.24 m^2^/g and was 1.50 nm.

#### Zeta potential

Figure 5D displays that the point of zero charges of ATP@Fe_3_O_4_-NH_2_Cs composite is 6.9. Thence at pH < 6.9; ATP@Fe_3_O_4_-NH_2_Cs surface is positively charged due to the protonation of NH_2_ groups, providing columbic interactions between the positive charges on the ATP@Fe_3_O_4_-NH_2_Cs composite surface and the negatively charged Cr(VI) ions. Contrariwise, beyond pH 6.9 ATP@Fe_3_O_4_-NH_2_Cs composite displays negative charges, causing electrostatic repulsion forces with the anionic Cr(VI).

### Removal of Cr (VI) by ATP@Fe_3_O_4_-NH_2_Cs composite

**Figure 6:**
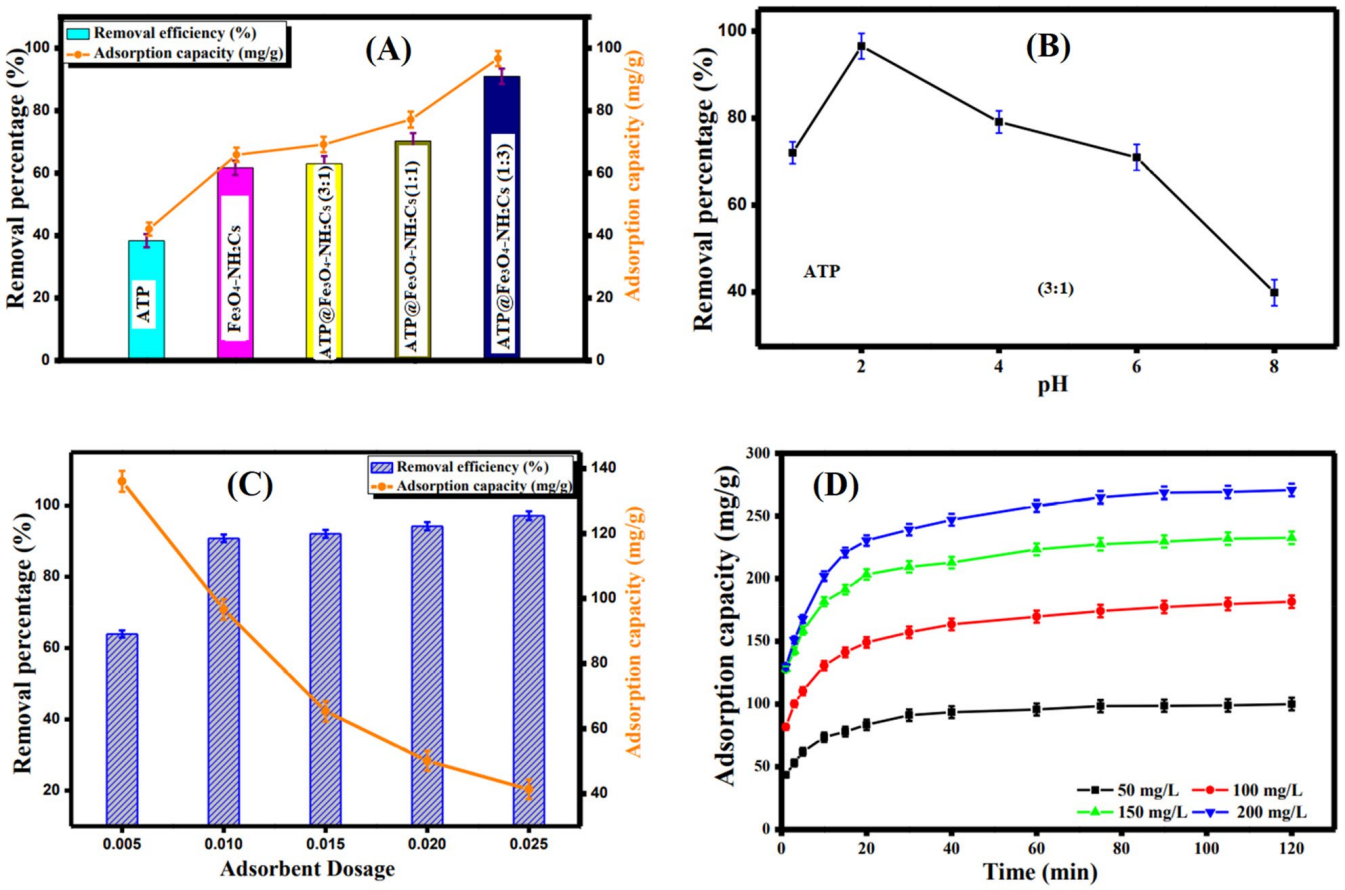
(**A**) Adsorption profiles of Cr(VI) onto ATP, Fe_3_O_4_-NH_2_Cs, ATP@Fe_3_O_4_-NH_2_Cs (3:1), ATP@Fe_3_O_4_-NH_2_Cs (1:1) and ATP@Fe_3_O_4_-NH_2_Cs (1:3), (**B**) Effect of pH, (**C**) Effect of dosage and (**D**) Effect of initial Cr(VI) concentration on the adsorption capacity.

A comparative test was executed to compare the efficacy of the different synthesized composites to determine which ratio has the finest adsorption capacity towards the Cr(VI) ions under the same adsorption conditions. Moreover, the same test was conducted for the pristine materials to trace the improvement in their adsorption behaviors after combination. Figure 6A showed that the adsorption capacity value increased in the order of ATP@Fe_3_O_4_-NH_2_Cs(1:3) (96.68 mg/g) > ATP@Fe_3_O_4_-NH_2_Cs(1:1) (77.14 mg/g) > ATP@Fe_3_O_4_-NH_2_Cs(3:1) (69.16 mg/g) > Fe_3_O_4_-NH_2_Cs (65.88 mg/g) > ATP (42.1 mg/g). These results confirm the improvement in the adsorption characters of the three composites comparing with the pristine as a result of the generated synergetic effect between Fe_3_O_4_-NH_2_Cs and ATP clay. On the basis of these results, the synergetic effect of ATP was calculated and listed in Table 1. It was obvious that the incorporation of ATP clay greatly enhanced the removal efficiency of Fe_3_O_4_-NH_2_Cs. Moreover, the increase in the adsorption capacity with increasing the Fe_3_O_4_-NH_2_Cs dosage in the adsorption medium is most likely due to increasing the surface positive charges resultant from the extra amine groups of aminated chitosan (NH_2_Cs). Accordingly, ATP@Fe_3_O_4_-NH_2_Cs was picked out for the subsequent studies.

**Table 1:** Synergetic effect of ATP clay to Fe_3_O_4_-NH_2_Cs in the removal of Cr(VI).

Sample code	Sample composition ATP : Fe_3_O_4_-NH_2_Cs	q_cal_ (mg/g)	q_exp_ (mg/g)	Synergetic effect (%)
ATP@Fe_3_O_4_-NH_2_Cs **1:3**	**(1:3)**	**59.94**	**96.68**	**61.30**
ATP@Fe_3_O_4_-NH_2_Cs **1:1**	**(1:1)**	**53.99**	**77.14**	**42.88**
ATP@Fe_3_O_4_-NH_2_Cs **3:1**	**(3:1)**	**48.05**	**69.16**	**44.00**

#### Effect of pH

Indeed, pH dominates the form of Cr(VI) in the aqueous solution, where Cr(VI) presents as H_2_CrO_4_ at pH = 1, while it exists as Cr_2_O_7_^2−^ and HCrO_4_^-^ at pH ranging from 2 to 6, however, CrO_4_^2−^ is the prime form at pH > 6^57^. In general, at high acidic medium NH_2_ groups protonate to NH_3_^+^, that charges ATP@Fe_3_O_4_-NH_2_Cs composite surface with a positive charge. It is apparent from Fig. 6B that the increase in pH from 1 to 2 results in an increase in the removal percentage from 65.53 to 90.81% and the adsorption capacity from 72.03 to 96.54 mg/g. This anticipated behavior can be assigned to the existence of Cr(VI) at pH = 1 in a neutral form (H_2_CrO_4_) which leads to a decrease in the columbic interactions between the cationic groups of ATP@Fe_3_O_4_-NH_2_Cs composite and the neutral H_2_CrO_4_ molecules^7^. However, at pH 2, there are resilient electrostatic interactions between the protonated NH_3_^+^ positive groups on the adsorbent surface and the negative charges of Cr(VI) species. One the other hand, beyond pH 2 there is an anticipated decrease in the number of protonated amine groups on the ATP@Fe_3_O_4_-NH_2_Cs surface. Consequently, the electrostatic interactions between ATP@Fe_3_O_4_-NH_2_Cs composite and Cr(VI) decreases, so the removal percentage and the adsorption capacity directly dwindle from 90.81% and 96.54 mg/g to 38.57% and 39.85 mg/g, respectively^58^. Based on these results, pH 2 was selected as an optimum pH value for the following adsorption studies.

#### Effect of ATP@Fe_3_O_4_-NH_2_Cs dosage

Figure 6C denotes the effect of adsorbent dosage on the adsorption profile. It is evident that increasing the ATP@Fe_3_O_4_-NH_2_Cs composite dosage from 0.005 to 0.025 g leads directly to a dramatically reduction in the adsorbed quantity of Cr(VI) from 136.07 to 41.34 mg/g, respectively, which may be attributed to the aggregation of ATP@Fe_3_O_4_-NH_2_Cs particles. Contrariwise, the Cr(VI) removal % was gradually increased from 63.17 to 97.17% with increasing the composite dosage as a result of increasing the adsorption active sites on the composite surface^59^.

#### Effect of initial concentration

Figure 6D points out that the adsorption capacity value significantly increases from 99.99 to 270.68 mg/g with increasing Cr(VI) concentration from 50 to 200 mg/L. These findings are expected due to increasing the driving forces that overcomes the mass transfer resistance of Cr(VI) ions from bulk to the ATP@Fe_3_O_4_-NH_2_Cs surface with increasing the initial Cr(VI) concentration. On the contrary, Figure (S1) shows a decline in the removal (%) value from 94.24 to 64.93% with rising the Cr(VI) concentration, which could be explained by the shortage of the adsorption active sites at constant adsorbent dosage^12^.

### Adsorption isotherms

To deduce the interaction sort between Cr(VI) and ATP@Fe_3_O_4_-NH_2_Cs composite, the obtained equilibrium data were scrutinized by bountiful isotherm models like Langmuir, Freundlich, Temkin and Dubinin-Radushkevich (D-R). The linearized isotherm equations are listed in Table S1^60,61^.

**Figure 7:**
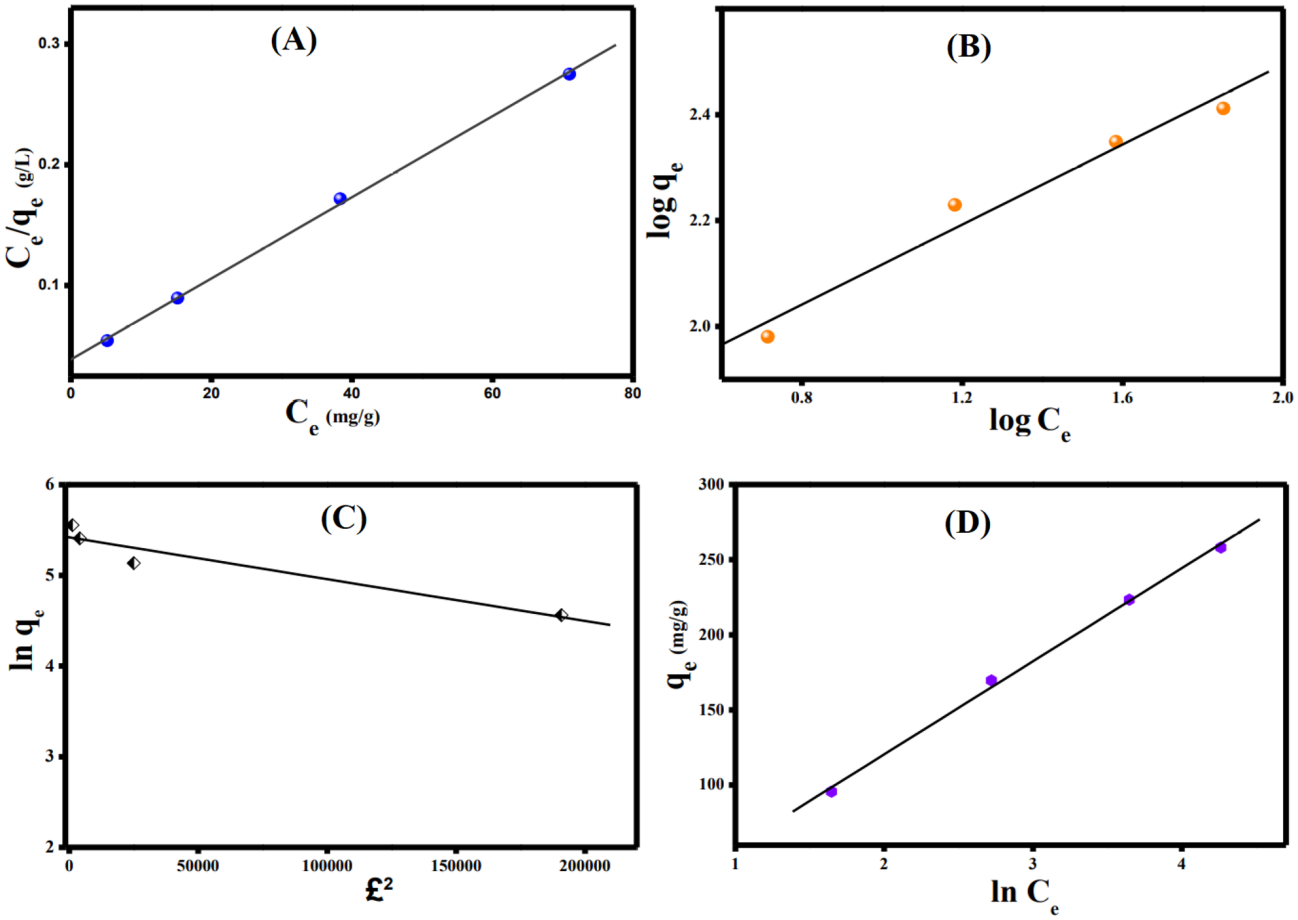
Isotherms plots for the Cr (VI) adsorption onto ATP@Fe_3_O_4_-NH_2_Cs composite; (**A**) Langmiur, (**B**) Freundlich, (**C**) Temkin, and (**E**) D-R.

The plots of the applied isotherm models are illustrated in Fig. 7A–D. It was inferred from R^2^ values (Table 2) that the inspected Cr(VI) adsorption process obeys Langmuir (0.999) and Temkin (0.998) more than Freundlich (0.953) and D-R (0.915).

**Table 2:** The parameters derived from isotherm models for the adsorption of Cr(VI) ions onto ATP@Fe_3_O_4_-NH_2_Cs composite.

Isotherm model	Parameter	Value
Langmuir	q_m_ (mg/g)	294.118
	b (L/mg)	0.088
	R^2^	0.999
Freundlich	n	2.646
	k_F_ (mg/g)(mg/L)^-1/n^	84.411
	R^2^	0.953
Temkin	A (L/g)	0.946
	B (J/mol)	61.948
	b (KJ/mol)	0.039
	R^2^	0.998
D-R	q_s_	225.56
	K_ad_ (mol^2^/K^2^J^2^)	5 × 10^–6^
	R^2^	0.915
	E (kJmol^-1^)	0.316

The computed Langmuir parameters clarify that q_max_ of Cr(VI) onto ATP@Fe_3_O_4_-NH_2_Cs composite is 294.12 mg/g, agreeing with the actual maximum adsorption capacity (270.68 mg/g). Moreover, Freundlich constant evinces the favorability of the Cr(VI) adsorption onto ATP@Fe_3_O_4_-NH_2_Cs composite at which n > 2. In addition, Temkin infers that the Cr(VI) ions adsorb onto ATP@Fe_3_O_4_-NH_2_Cs composite via physical adsorption, agreeing with D-R model result since the calculated bonding energy ($\mathrm{E}=\frac{1}{\sqrt{2{\mathrm{K}}_{\mathrm{ad}}}})$ < 8 kJmol^-1^. In general, physical adsorption takes place via weak Van der Waals interactions, so the Cr(VI) adsorption onto ATP@Fe_3_O_4_-NH_2_Cs composite requires a low adsorption energy^62^.

### Adsorption kinetics

**Figure 8:**
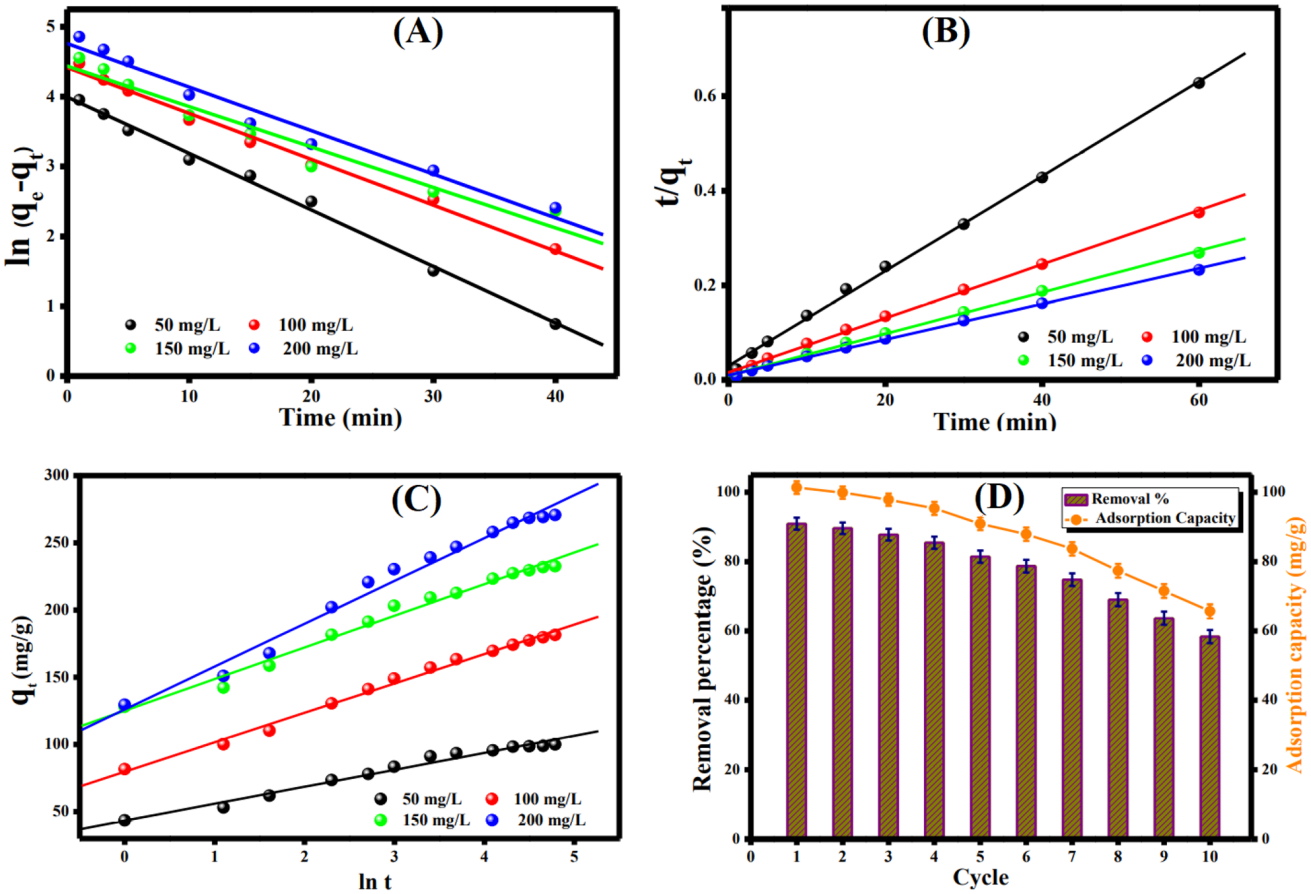
Kinetic plots for the Cr(VI) adsorption onto ATP@Fe_3_O_4_-NH_2_Cs composite; (**A**) Pseudo 1st order, (**B**) Pseudo 2nd order, and (**C**) Elovich and (**D**) Regeneration and reusability of ATP@Fe_3_O_4_-NH_2_Cs composite.

The Cr(VI) adsorption mechanism of onto ATP@Fe_3_O_4_-NH_2_Cs composite was identified utilizing Pseudo 1st order, Pseudo 2nd order and Elovich (Fig. 8A–C). The linearized kinetic equations were summarized in Table S2.

It was concluded that the Cr(VI) adsorption process onto ATP@Fe_3_O_4_-NH_2_Cs best fits pseudo 2nd order based on the R^2^ values (Table 3). Also, the computed q values from pseudo 2nd order seem to resemble the experimental values, evincing the suitability of pseudo 2nd order to represent the studied adsorption process. In addition, it was noticed a decline in the k_2_ values with the rising in the Cr(VI) initial concentration, suggesting the chemical adsorption process of Cr(VI) onto ATP@Fe_3_O_4_-NH_2_Cs composite^59^. Moreover, the computed Elovich coefficients elucidate that the rate of Cr(VI) adsorption is vaster than the desorption since the α values exceed the β values^63^.

**Table 3:** Adsorption kinetic model parameters of the adsorption of Cr(VI) onto ATP@Fe_3_O_4_-NH_2_Cs composite.

Kinetic models and parameters		Concentration (mg/L)						
		50		100		150		200
q_e, exp_(mg/g)		99.99		181.51		232.56		270.68
**Pseudo 1storder**								
q_e,cal_(mg/g)		54.36		82.76		84.50		116.69
k_1_(min^-1^)		0.081		0.066		0.058		0.062
R^2^		0.995		0.992		0.954		0.971
**Pseudo 2ndorder**								
q_e,cal_(mg/g)		100		175.44		227.27		263.16
k_2_(g.mg^-1^.min^-1^)		0.003		0.002		0.001		0.0009
R^2^		0.998		0.998		0.999		0.999
**Elovich**								
α (mg/g min)		388.97		834.27		4783.07		1658.44
β (g/mg)		0.0792		0.0457		0.425		0.0314
R^2^		0.979		0.993		0.985		0.983

### Thermodynamics

To assess the impact of change the reaction temperature on the nature of Cr(VI) adsorption process onto ATP@Fe_3_O_4_-NH_2_Cs composite, the thermodynamics parameter such as, change in entropy (ΔSº), change in enthalpy (ΔHº) and change in free energy (ΔGº) were reckoned from Eqs. 3 and 4.

3 $${\mathrm{lnK}}_{\mathrm{e}}=\frac{\Delta {\mathrm{S}}^{\mathrm{o}}}{\mathrm{R}}-\frac{\Delta {\mathrm{H}}^{\mathrm{o}}}{\mathrm{RT}}$$

4 $${\Delta \mathrm{G}}^{\mathrm{o}}={\Delta \mathrm{H}}^{\mathrm{o}}-\mathrm{T}{\Delta \mathrm{S}}^{\mathrm{o}}$$

where, ${K}_{e}=\frac{{C}_{Ae}}{{C}_{e}}$ is the thermodynamic equilibrium constant; C_e_ and C_Ae_ are the Cr(VI) concentration in the solution and onto ATP@Fe_3_O_4_-NH_2_Cs surface at equilibrium, respectively. R and T are gas constant and adsorption temperature, respectively.

The computed thermodynamics parameters demonstrate that the Cr(VI) adsorption onto ATP@Fe_3_O_4_-NH_2_Cs is randomness and endothermic process owing to the positive values of both ∆S^o^ and ∆H^o^ that have been reckoned from Van't Hoff Plot (Figure S2). Also, ∆H^o^ value suggesting the chemical adsorption process since it falls between 40 and 200 kJ/mol^64^. Besides, the negative values of ∆G^o^ (Table 4) elucidate the spontaneity of the Cr(VI) adsorption onto ATP@Fe_3_O_4_-NH_2_Cs^59^.

**Table 4:** Thermodynamic parameters of the adsorption of Cr(VI) onto ATP@Fe_3_O_4_-NH_2_Cs composite.

ΔG°(kJ/mol)				ΔH° (kJ/mol)	ΔS° (J/mol K)
298 K	308 K	318 K	328 K	8.45	46.77
− 13.98	− 14.40	− 14.86	− 15.33		

### Reusability

To evaluate the ability of ATP@Fe_3_O_4_-NH_2_Cs magnetic composite to reuse for several adsorption cycles, ten successive adsorption–desorption processes were executed. Figure 8D points out that the developed adsorbent still retains respectable adsorption characteristics even after 10 cycles with maximum adsorption capacity of 62.54 mg/g and maximum Cr(VI) removal % of 58.36%, indicating the well-recycling property of the as-fabricated ATP@Fe_3_O_4_-NH_2_Cs magnetic composite. This fascinating behavior to the as-fabricated ATP@Fe_3_O_4_-NH_2_Cs composite is may be due to its magnetic property that provides a perfect separation to the composite after the adsorption process without losing in its mass.

### Comparison with other studies

To assess the developing behavior of our novel adsorbent, a comparison study was executed between the as-fabricated ATP@Fe_3_O_4_-NH_2_Cs composite and other reported adsorbents in previous studies (Table 5). Notably, ATP@Fe_3_O_4_-NH_2_Cs composite has a dual function; an excellent adsorption capacity towards Cr(VI) ions (294.12 mg/g) and a fast adsorption process as it reaches equilibrium at about one hour. This fascinating adsorption property of ATP@Fe_3_O_4_-NH_2_Cs composite is most likely due to the synergistic effect between ATP and Fe_3_O_4_-NH_2_Cs. Over and above, the extra- amine groups boost the cationic nature of the composite surface, and consequently causing more electrostatic interactions between the anionic Cr(VI) and the positively charged surface of ATP@Fe_3_O_4_-NH_2_Cs composite.

**Table 5:** Comparison of the maximum adsorption of Cr(VI) with numerous adsorbents.

Adsorbent	q_max_ (mg/g)	Equilibrium time (min)	References
Chitosan nanofibers	131.58	480	^70^
Malic acid-chitosan beads	382.20	120	^71^
MAC–attapulgite composite	119.62	120	^72^
Attapulgite-supported nZVI composite	266.65	720	^73^
Polypyrrole/molybdenum disulfide composite	257.73	900	^74^
bentonite@MnFe_2_O_4_composite	161.30	90	^75^
Polyaniline**/**Bi(III) iodomolybdate composite	240.90	40	^76^
BM-FeS_2_@BC700	134.00	100	^77^
MoS_2_@LDC) composite	198.70	40	^78^
Fe_3_O_4_/ZIF-67@AmCs beads	119.05	80	^65^
ATP@Fe_3_O_4_-NH_2_Cs composite	294.12	60	This work

### Adsorption mechanism

**Figure 9:**
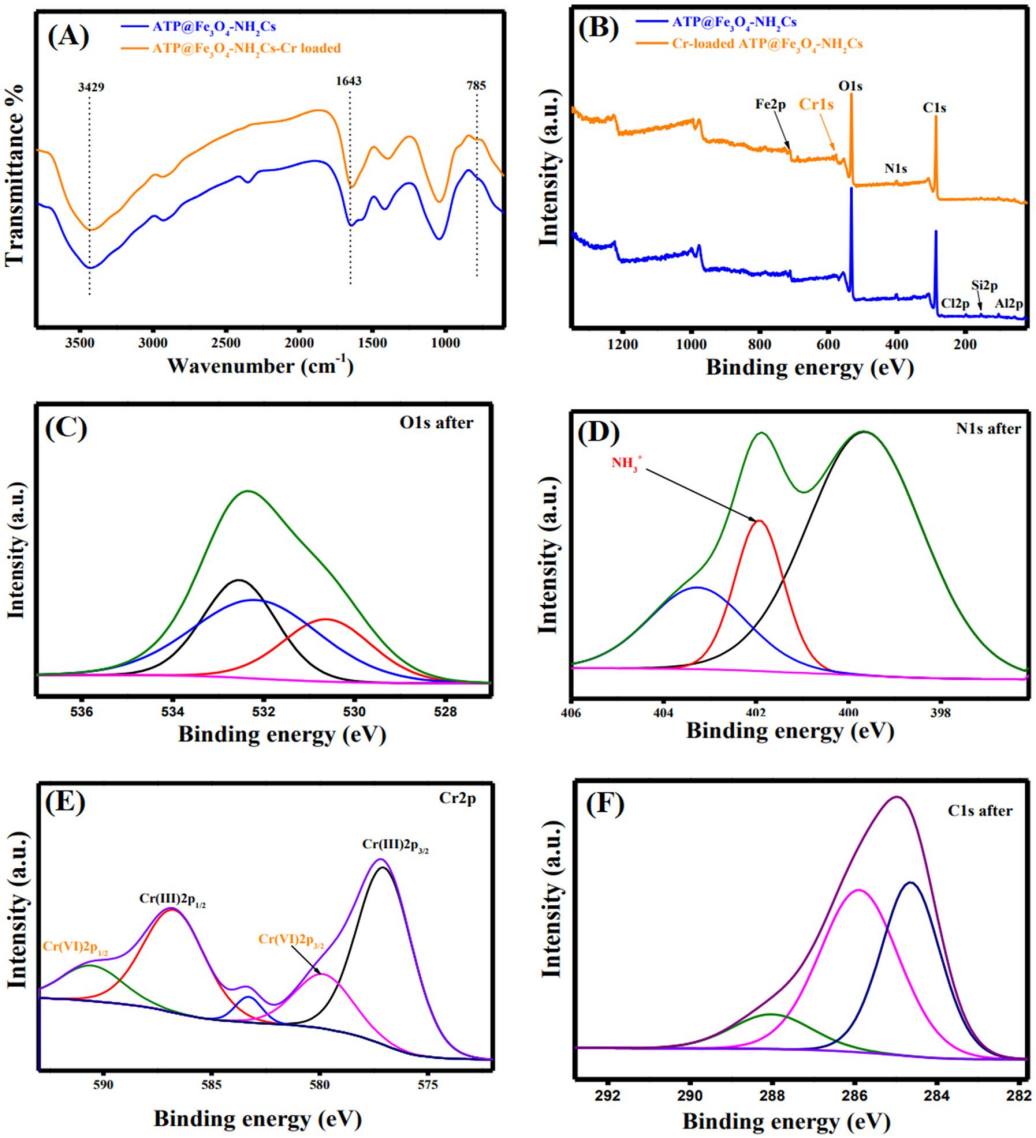
(**A**) FTIR of ATP@Fe_3_O_4_-NH_2_Cs composite before and after adsorption of Cr(VI), (**B**) XPS spectra of ATP@Fe_3_O_4_-NH_2_Cs before and after adsorption of Cr(VI), (**C**) Cr2p, (**D**) N1s, (**E**) O1s and (**F**) C1s after adsorption of Cr(VI) onto ATP@Fe_3_O_4_-NH_2_Cs composite.

**Figure 10:**
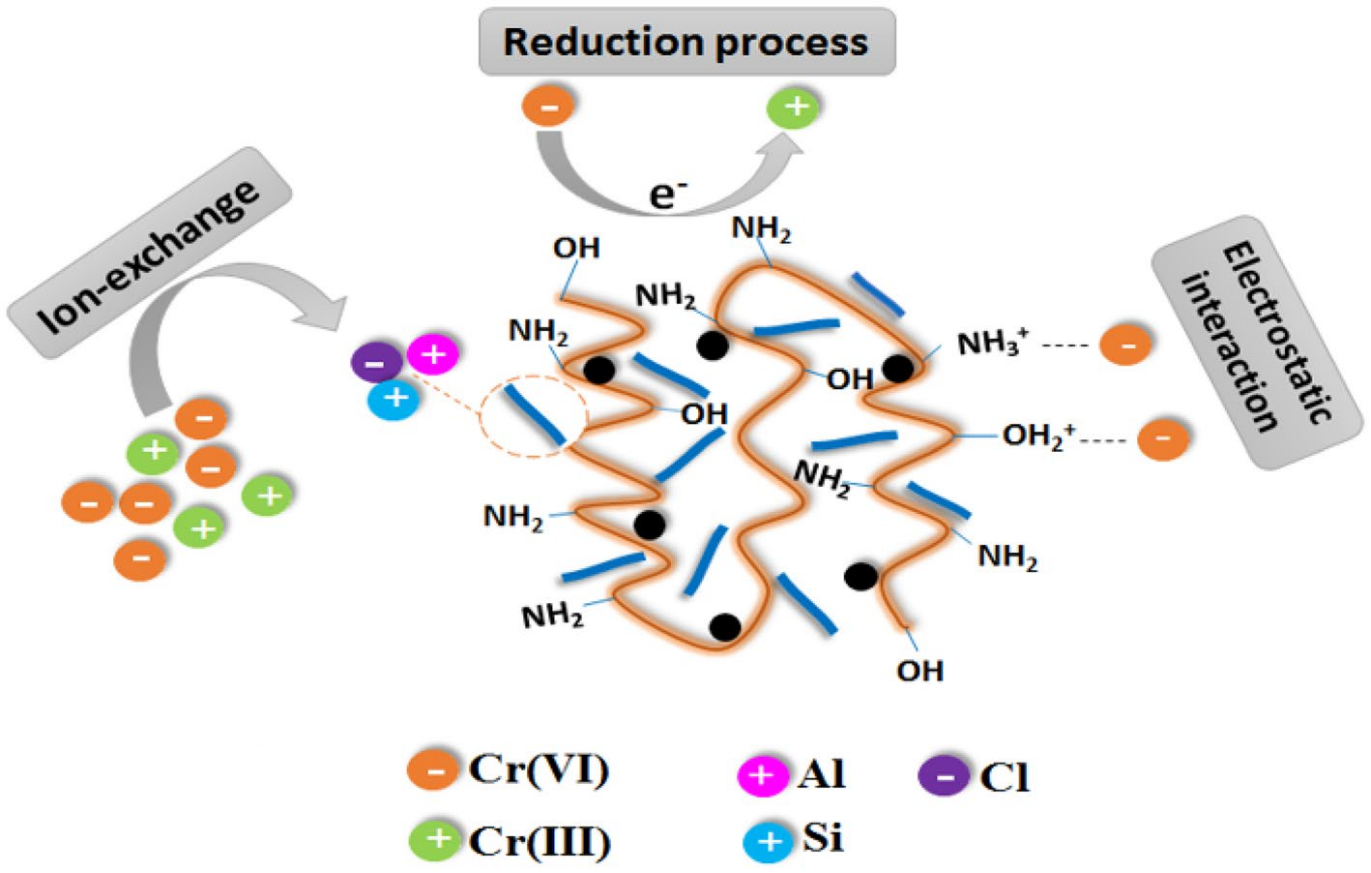
Proposed mechanism for removal Cr(VI) onto ATP@Fe_3_O_4_-NH_2_Cs composite.

The mechanism of Cr(VI) adsorption onto ATP@Fe_3_O_4_-NH_2_Cs adsorbent was deduced depending on the gained FTIR and XPS data before and after the adsorption process. FTIR spectrum of ATP@Fe_3_O_4_-NH_2_Cs composite after adsorption of Cr(VI) (Fig. 9A) illustrates the distinctive peak of tetrahedron CrO_4_ at 785 cm^−1^^65^. Besides, the bands related to both of –NH_2_ and –OH^−^ groups are moved from 1643 and 3429 cm^−1^ to 1639 and 3424 cm^−1^, respectively, implying that hydroxyl and amine groups of ATP@Fe_3_O_4_-NH_2_Cs composite are involved in the adsorption of Cr(VI) ions^66^. Moreover, XPS spectrum of ATP@Fe_3_O_4_-NH_2_Cs composite after the adsorption process (Fig. 9B) reveals the belonging peak to Cr2p at BE of 578.16 eV. In addition, the results of Fig. 9C approve that the adsorption process occurs via reduction of Cr(VI) to Cr(III) at which the characteristics peaks of Cr(III) 2p_1/2_ and Cr(III) 2p_3/2_ emerged at BE of 586.75 and 577.01 eV, respectively^66^. While, the peaks at BE of 592.98 and 579.82 eV are related to Cr(VI) 2p_1/2_ and Cr(VI) 2p_3/2_, respectively. Furthermore, there is a decline in the intensity of Al2p, Si2p and Cl2p peaks which may be interpreted by the partial exchange of attapulgite metal ions with Cr(VI) and Cr(III), suggesting the ion exchange mechanism^47,67^. On the other hand, Fig. 9D shows the peaks at BE of 398.38 and 400.16 eV which are ascribed to –NH_2_ and NH groups, respectively. Whereas, the spectrum of N1s after the adsorption of Cr(VI) infers the protonation of the amine group at low pH, since the distinctive peak of NH_3_^+^ appeared at BE of 402 eV, suggesting the possibility of electrostatic interaction mechanism between the anionic Cr(VI) ions and NH_3_^+^ on the surface of the composite. Furthermore, the wide-spectrum of O1s after the adsorption process (Fig. 9E) clearly revealed a decrease in the intensity of OH and Si–O–Si peaks which could be attributed to exchange of OH and Si with Cr(VI) and Cr(III) ions^68^. In addition, it was found a slight shift around 0.3 eV in the binding energy of C1s after the adsorption of Cr(VI) (Fig. 9F) which may be ascribed to the reaction of Cr(VI) ions with oxygen and nitrogen function groups, agreeing with FTIR results^69^. To sum, FTIR and XPS results suppose that mechanism of Cr(VI) adsorption onto ATP@Fe_3_O_4_-NH_2_Cs composite involve the electrostatic interactions, reduction of Cr(VI) to Cr(III) and ion-exchanging (Fig. 10).

## Conclusion

In this study, ATP@Fe_3_O_4_-NH_2_Cs composite was formulated with different proportions for efficient adsorption for Cr(VI) ions from their aqueous solutions. The utilized characterization tools elucidated the good thermal and magnetic characteristics of the as-fabricated ATP@Fe_3_O_4_-NH_2_Cs composite in addition to its higher surface area. Furthermore, batch adsorption experiments clarified that the best adsorption capacity values were attained at pH 2 and achieved by ATP@Fe_3_O_4_-NH_2_Cs(1:3). Moreover, isotherms studies revealed an analogy between the calculated maximum adsorption capacity under Langmuir isotherm model (294.12 mg/g) and the experimental one (270.68 mg/g). In addition, kinetics studies validated that the adsorption process follows the pseudo 2nd order kinetic model, while the thermodynamic parameters recognized the process to be endothermic, spontaneous and randomness. Furthermore, the results assumed that the adsorption process of Cr(VI) ions occurred via the electrostatic interaction between opposite charges, reduction of Cr(VI) to Cr(III) and ion-exchanging mechanisms. Finally, reusability test proved also the excellent potential of ATP@Fe_3_O_4_-NH_2_Cs adsorbent composite to be reuse for several times, which is a beneficial for its application for removing of Cr(VI) ions from contaminated water. It can be concluded that the as-fabricated ATP@Fe_3_O_4_-NH_2_Cs composite could be applied as sustainable and reusable adsorbent for removing Cr(VI) ions from wastewater.

## Supplementary Information

Supplementary Information.
