# Supplementary material for: Fabrication of attapulgite/magnetic aminated chitosan composite as efficient and reusable adsorbent for Cr (VI) ions
Source: Sci Rep. 2021 Aug 16;11:16598. doi: 10.1038/s41598-021-96145-6 (PMC8368087; doi:10.1038/s41598-021-96145-6)
Supplement: Supplementary file 1 — Supplementary Information. [file 41598_2021_96145_MOESM1_ESM.docx]

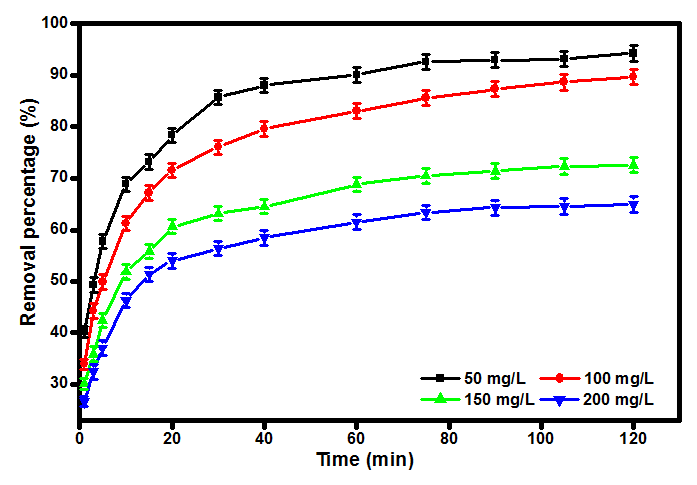


**Figure S1.** Effect of initial concentration on the removal (%) of Cr(VI) onto ATP-mNH_2_Cs1.5 composite [pH= 2, dose= 0.5 g/L and T= 25 ºC].

**
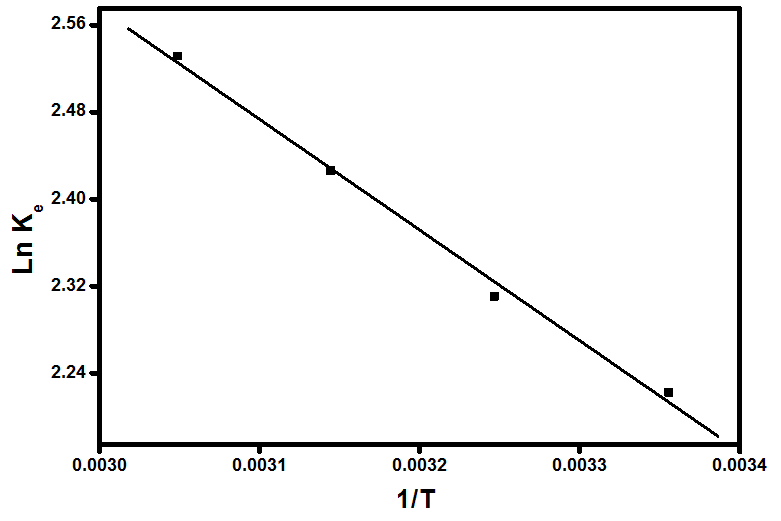
**

**Figure S2.** Van't Hoff plot of adsorption of Cr(VI) onto ATP-mNH_2_Cs composite.

**Table S1.** Equations of the applied adsorption isotherm models.

| **Model** | **Equation** |
| --- | --- |
| **Langmuir** | $\frac{C_{e}}{q_{e}}=\frac{1}{K_{L} q_{m}}+\frac{C_{e}}{q_{m}}$ (3) |
| **Freundlich** | $\log q_{e}=\log K_{F}+\frac{1}{n}\log C_{e}$(4) |
| **Temkin** | $q_{e}=B lnA+B\ln C_{e}$ (5) |
| **D-R** | $\mathrm{Ln}q_{e}=Ln q_{s}-K_{\mathrm{ad}}\varepsilon^{2}$,$\varepsilon=RT Ln \left( 1+\frac{1}{C_{e}} \right)$ $(6-7)$ |

Where, q_e_ and C_e_ are the adsorption capacity and the concentration of the un-adsorbed Cr(VI) at equilibrium, respectively. q_m_ and K_L_ are the monolayer adsorption capacity and Langmuir constant, respectively. n and K_F_ are Freundlich constants. A is the equilibrium binding constant and $B=\frac{\mathrm{RT}}{b}$ , b is Temkin constant related to heat of adsorption. R is the gas constant (8.314 J/mol.k) and T is the absolute temperature. q_s_ is the saturation capacity, ε is the Polanyi potential and K_ad_ is a constant related to the mean free energy of adsorption per mole of the adsorbate.

**Table S2.** Equations of the applied adsorption kinetic models.

| **Kinetic Model** | **Equation** |
| --- | --- |
| **Pseudo 1^st^ order** | $\ln(q_{e}-q_{t})=\ln q_{e}-k_{1}\left( t \right) (8)$ |
| **Pseudo 2^nd^ order** | $t/{q_{t}}=1/{k_{2}q_{e}^{2}+ 1/{q_{e}}\left( t \right) (9)}$ |
| **Elovich model** | $q_{t}=\frac{1}{\beta}\ln\left( \alpha\beta\right)+ \frac{1}{\beta}$ ln (t) (10) |

Where, q_t_ and q_e_ are amount of Cr(VI) adsorptions at time t and equilibrium, respectively. k_1_ and k_2_ are the rate constants of pseudo 1^st^ order and pseudo 2^nd^ order, respectively. α and β are Elovich coefficients that represent the initial adsorption rate and the desorption coefficient, respectively, also related to the extent of surface coverage and activation energy for chemisorption.
